# Using the Public Involvement Impact Assessment Framework to assess the impact of public involvement in a mental health research context: A reflective case study

**DOI:** 10.1111/hex.12688

**Published:** 2018-04-25

**Authors:** Michelle Collins, Rita Long, Anthony Page, Jennie Popay, Fiona Lobban

**Affiliations:** ^1^ Division of Health Research Lancaster University Lancaster UK; ^2^ Spectrum Centre Lancaster University Lancaster UK

**Keywords:** impact assessment, Public involvement impact Assessment Framework, Public Involvement in research, reflective case study

## Abstract

**Background:**

We assess the utility of the Public Involvement Impact Assessment Framework (PiiAF) as a resource to support research teams in assessing the impact of Public Involvement across diverse research and public involvement (PI) contexts. PiiAF was developed in response to a well‐documented growth in Public Involvement in health research in the United Kingdom that demands a more sophisticated evidence base to demonstrate its impact.

**Design:**

We used a reflective case study approach drawing on contemporaneous meeting notes, PiiAF website resources and retrospective reflections to describe how PiiAF helped us to develop an impact assessment plan of the PI in a university‐based mental health research centre.

**Discussion:**

We consider key aspects of our experiences of using PiiAF as a tool to help us design an impact assessment of PI, interpret these experiences with reference to relevant theory and research and share insights that may be useful to other teams considering using PiiAF.

**Conclusion:**

These insights include understanding the commitment of time and effort required to develop effective PI impact assessment plans; the flexibility of PiiAF and its ability to be used in a range of research and PI contexts; and the advantages of involving all stakeholders (including the public) in the development of an PI assessment plan.

## 1 INTRODUCTION

This paper reports on the utility of the Public Involvement Impact Assessment Framework (PiiAF) by describing our experience of the PiiAF process in developing a plan to assess the impact of public involvement (PI) in a mental health research centre.

The different terminologies which are used to describe PI presented a potential challenge for us in writing this paper. The team involved in developing PiiAF originally took a deliberate decision to use the term “Public Involvement” in an inclusive way to refer to all forms of public, patient, service user, community involvement in research.^1^ In the mental health setting in which this particular study took place, PI is primarily focused on involving people with lived experience of mental health problems and their relatives.

### 1.1 Background to the development of PiiAF

The growth of PI in health research in the United Kingdom in particular is well documented; however, some concerns about the sophistication of the evidence regarding its impact have been articulated.^2^, ^3^ As the field matures, debates around PI reflect both the complexity of the underpinning theoretical and conceptual issues along with the practical challenges of involving members of the public in health research contexts. There is broad agreement that the public have a right to be involved in research related to health conditions or issues related to them.^4^, ^5^, ^6^ However, there are also advantages to developing an evidence base regarding the impact of PI on a range of potential outcomes.^1^ Firstly, by exploring the relative effectiveness of different PI approaches, we can begin to understand the mechanisms by which PI can impact on research and consequently develop more effective involvement strategies, increasing the value of PI for both the public and researchers. Secondly, health research funders require detailed justification and costing for all elements of research including PI. Evidence to demonstrate the effectiveness and value for money of PI would strengthen the rationale for this funding.

Many models and frameworks exist to describe the different ways in which PI can happen^7^, ^8^, ^9^ and to assess its quality.^10^ Reviews of the PI literature have concluded that despite the limited nature of the evidence base, and lack of explicit conceptualizations of the PI underpinning the research, a wide variety of PI impacts can be identified.^2^, ^11^ But there are fewer attempts to demonstrate how the impact can be directly assessed (for exceptions see^2^, ^3^). PiiAF addresses some of these concerns. It seeks to support people to identify the types of impact they might reasonably expect for PI in specific research projects/programmes provides a new framework for assessing PI impacts and guidance on how to do this.

PiiAF is based on research carried out by a multidisciplinary team of researchers and members of the public across the United Kingdom, funded by the Medical Research Council's NIHR Methodology Research Programme (Grant No G0902155/93948). The framework was developed through 3 phases: an evidence review of the values and impacts of PI in health and social care research;^12^ a Delphi survey of key PI stakeholders around consensus and conflict in PI;^13^, ^14^ and development and initial testing of the PiiAF.^1^

Members of the public played a key part in the development of PiiAF. This included the involvement of service user investigators with experience of facilitating PI in research in developing the proposal, as members of the research team and in the project's management group; a Public Advisory Group comprising people with experience of being involved in health research, which met 5 times and commented on all aspects of the research; and an Advisory Network, including members of the public as well as academics and professionals with experience of PI which met 3 times and advised on the strategic development and delivery of the project. A systematic internal formative evaluation of the process and impacts of PI in the research project was also undertaken.

PiiAF conceptualizes PI in research as a complex social process and acknowledges the significance of the personal and organizational contexts in which it takes place as well as the difficulties associated with attributing specific impacts to PI in general or to specific aspects of the PI process.^1^ Consequently, PiiAF promotes an explicit values‐based approach to PI and its assessment that focuses amongst other things, on understanding the motivations for researchers and members of the public to engage in PI, which is considered important in increasing the likelihood of effective PI and positive outcomes. PiiAF does not therefore provide a quick‐fix tool or measure of PI impact, but rather supports research teams (including public advisers) through a developmental process, the aim of which is to design a plan to assess PI impact that will meet the needs of their specific project.

PiiAF was primarily intended to be used by research teams for PI impact assessment planning in the initial development of single research project funding proposals. The framework however was also designed to be flexible and to be applied to the range of different contexts in which PI in research happens. This could include, for example, plans to assess the impact of PI priority setting in programme grant applications.

Two members of the team that developed PiiAF, (FL and MC) along with 2 other colleagues wanted to test the utility of PiiAF in developing a plan to assess the impact of PI in a mental health research centre. In the next section, we briefly describe the structure of PiiAF and then go on to provide an overview of the research context in which we used PiiAF.

### 1.2 The PiiAF framework

**Figure 1:**
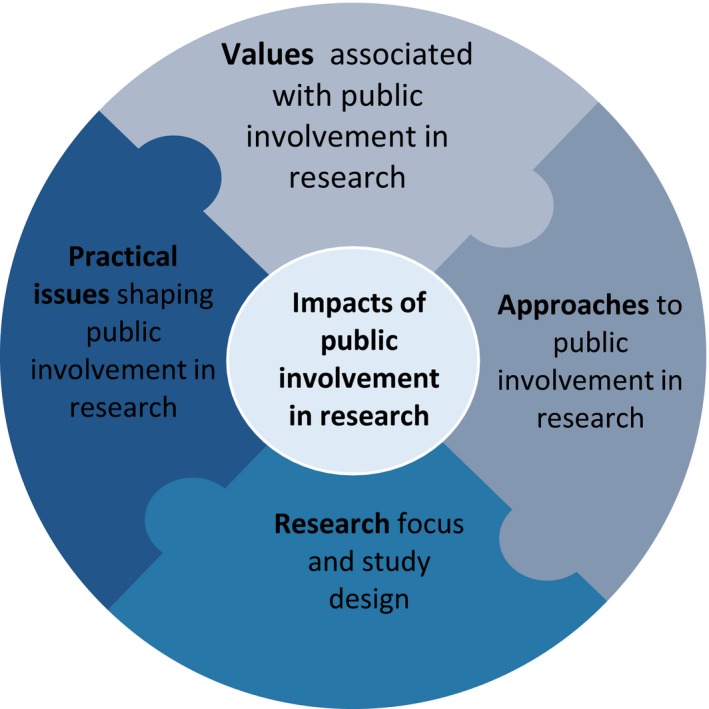
Part 1 of Public Involvement Impact Assessment Framework showing the 5 elements

PiiAF is structured into 2 parts. Part 1 (see Figure 1) highlights 5 elements that research teams might need to consider when planning their PI impact assessment: values associated with PI in research, approaches to PI, research focus and study design, practical issues shaping PI in research and identifying feasible Impacts. A series of key issues and questions accompany each element, inviting research teams to engage in an assessment process. The PiiAF and resources to support its use can be found at the following website.^1^

**Figure 2:**
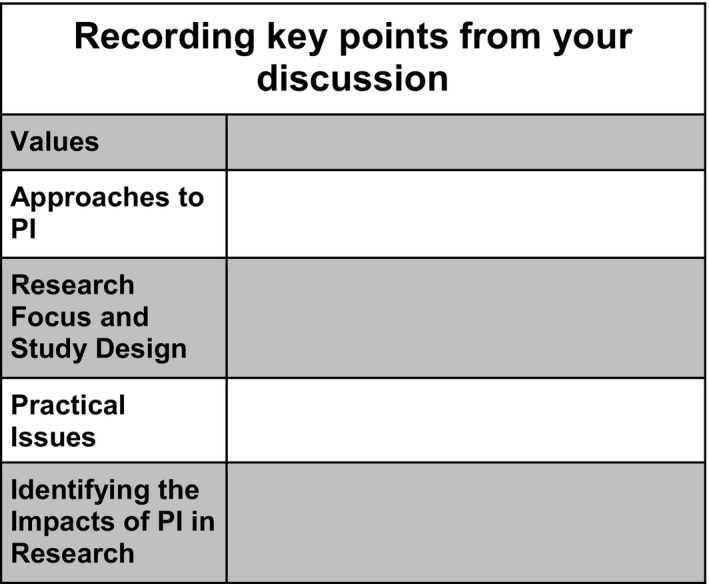
The record card

Responses to the issues and questions raised in each element of phase 1 can be captured and recorded using the Record Card which is a key PiiAF resource and can be obtained from the website^1^ (Figure 2).

**Figure 3:**
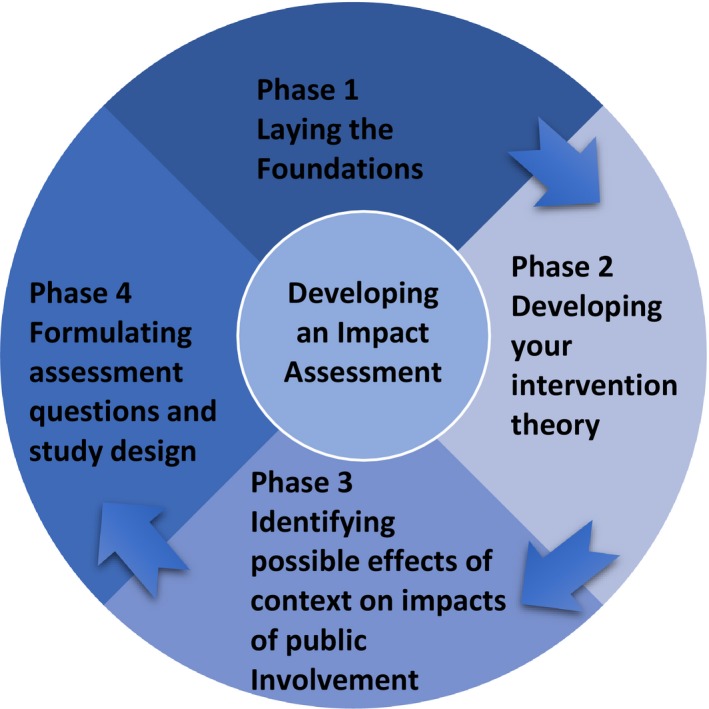
Part 2 of Public Involvement Impact Assessment Framework showing the 4 phases

Once completed, the record card can be used in Part 2 of the PiiAF process (see Figure 3) which guides research teams through 4 steps to create an impact assessment plan. These include working out why you want to assess the impact of PI and who should be involved in this work; developing your intervention theory (ie, showing how your approach to PI will lead to the desired impacts); identifying how the potential context of your research might affect the impact of PI; and finally utilizing all these features to formulate an impact assessment plan including the design of the assessment.

In the next section, we describe the research context in which we used the PiiAF process discussed above.

### 1.3 The Spectrum Centre for Mental Health Research

The Spectrum Centre for Mental Health Research was set up in 2008 at Lancaster University in the United Kingdom. At the time of the work reported here, the core funded team consisted of 2 professors, 3 lecturers, 2 part‐time service user researchers (SUR) and a part‐time administrator. There were also additional doctoral and post‐doctoral fellows and research associates within the team who were funded externally. The Spectrum Centre focuses on developing and evaluating psychosocial approaches to supporting people with severe mental health problems and their support networks (see Box 1).

BOX 1 **Public Involvement in the The Spectrum Centre**

1 The “public” involved in The Spectrum Centre work are primarily people with mental health problems (most commonly referred to as “service users”) as well as members of their support networks including carers, relatives and peer groups. Frontline clinical staff and health service commissioners are also key stakeholders in The Spectrum Centre. The work reported here focused specifically on developing a plan to assess the impact of involvement of service users in the Spectrum Centre's work. One of the authors (RL) was a service user research (SUR) responsible for co‐ordinating service user involvement across all Spectrum Centre research. RL chairs an Advisory Panel of 10‐12 service users and members of their support network, who meet bimonthly to discuss design, implementation and dissemination of new and ongoing research projects, involvement strategies in those projects and input to the strategic direction of the centre. Funding for this Advisory Panel activity is obtained through income gained from external teaching and examining, and fund‐raising activities. In addition to the Advisory Panel, grant funded studies include funding for study‐specific Service User Reference Groups. These are formed through formal and informal links and by advertising opportunities to members of Spectrum connect, a database of people who have consented to be kept informed about activities at the Spectrum Centre Centre consisting mainly of people who have taken part in research previously, although anyone can join.^2^

### 1.4 Study aims

Our aim was to capture our experience of using PiiAF to develop a plan to assess the impact of the range of methods of involving users of mental health services in the research carried out in the Spectrum centre and to share key aspects of this experience, which we hope, will be of use to others wishing to use PiiAF. To achieve this, we used a Reflective case study method.

## 2 METHOD

### 2.1 Methods used for capturing and reporting on our experiences of using PiiAF

We convened a Steering Group (SG) to oversee the use of PiiAF in the Spectrum Centre, which included FL (Co‐Director of Spectrum) and MC (Research Associate working in the same Division as Spectrum) who were both involved in the development of PiiAF, RL the SUR working in Spectrum mentioned earlier and finally AP, a Spectrum volunteer with a background in psychiatry and sociology who had no previous involvement with PiiAF. At initial meetings of the SG, we decided who should be involved in developing the PI impact assessment plan for the centre and familiarized ourselves with PiiAF and the processes involved in using it. The SG developed a protocol for the work (see Appendix 1) and met over a period of 9 months approximately every 3‐4 weeks initially and then less frequently to review and reflect on our actions and activity and to agree on next steps between January and September 2014.

We decided to use critical reflection as a method of capturing our experiences of using PiiAF. There are many models of reflective practice, 15 but all involve some way of making visible experiences, knowledge or actions that might otherwise remain hidden with the aim of informing future action. Boote et al's^16^ paper which is relevant to our work described PI in the selection of research ideas for development into grant applications and used a critical reflection framework based on Marks‐Maran and Rose.^17^ We used this critical reflection framework to capture and make visible our experiences of using PiiAF.

The Marks‐Maran & Rose (1997) framework is structured so that it enables us to (i) describe what happened when we implemented the PiiAF development process; (ii) to explore what we thought and felt about this process; (iii) to locate our experiences in the context of relevant previous research and (iv) to identify key learning points to take forward.

In applying the Marks‐Maran & Rose reflective framework to capture our experiences of using PiiAF, we drew upon a number of sources of information:

1. Contemporaneous SG meeting notes: minutes of SG meetings.
2. Outputs from phase 1 and phase 2 of the PiiAF process, including the information captured on the PiiAF record card at different stages of the PiiAF process. (see Appendix 2 for a copy of record card that we completed).
3. Retrospective structured reflections from the SG members. Each member of the SG was asked to complete a critical reflection template for each part of the PiiAF developmental process. We designed this template as a way of capturing retrospective reflections from the SG on key moments of the PiiAF process. The template consisted of a timeline of activities undertaken by the SG as part of the PiiAF impact assessment design process, including:
  - The initial SG meeting to discuss the work.
  - Administration of the audit tool to members of staff and members of the public involved in the centre's work to capture their values for PI for Part 1 of the PiiAF process.
  - Holding a workshop for centre staff members and members of public involved in the centre's work to develop a further understanding of responses to the audit tool.

These activities are described in greater detail below.

The critical reflection template invited SG members to reflect on what worked well or worked less well and how well they understood each part of the process. A blank template can be found in Appendix 3.

The following 4 sections of the paper draw on data recorded by the authors during the PiiAF process. Using the Marks‐Maran & Rose critical reflection framework, we will (i) describe our experience of using the PiiAF; (ii) critically reflect on key moments in the PiiAF process; (iii) locate our experience of using PiiAF in the context of existing PI impact assessment and literature; and finally (iv) share some insights for others who may wish to use the PiiAF in future.

## 3 OUR EXPERIENCES OF USING PIIAF DESCRIBED THROUGH THE 4 STAGES OF THE MARKS‐MARAN & ROSE CRITICAL REFLECTION FRAMEWORK

### 3.1 Description of our application of the PiiAF Process

The PiiAF process has 2 parts. Below we describe the activities that we undertook during Part 1 and Part 2 of the process.

#### 3.1.1 Using Part 1 of PiiAF

The main tasks in Part 1 of PiiAF are to identify the range of issues that researchers could consider when planning their PI and thinking about its impact. The SG intended to capture the values of as many Spectrum centre members as possible, as these are all likely to impact on the way PI happens in the centre. The SG developed and administered an audit tool designed to provide a systematic and independent examination of PI processes operating in the centre.

##### Audit tool

The audit tool questions were based on the PiiAF record card and invited respondents to identify the purpose, processes, impacts, context and assessment of PI in The Spectrum Centre. Questions required short open text responses, and a copy of this tool is available from the authors. The audit tool was not piloted because it was developed to reflect the PiiAF record card categories, which in turn was developed as part of PiiAF which was based on extensive underpinning research. Further, the audit tool was not intended to be a research tool, but a way of capturing values around PI in the centre. Finally, the number of people with whom we could use the tool was relatively small, and piloting the tool would have reduced the population still further.

The audit tool was emailed to staff members in the Spectrum Centre (n = 15) and all members of the Service User Advisory Panel (n = 12). Members of the Spectrum centre Advisory Panel also had an opportunity to complete this audit tool during one of their bimonthly meetings held in January 2014. Seventeen responses were returned (63%) (12 [70%] staff members, and 12 [30%] Advisory Panel members). Although we have no data on whether the PI values are of those who completed the audit tool were different from those who did not complete the tool, we are satisfied that we got a wide enough range of responses to make it a valuable exercise. Furthermore, the PiiAF process is intended to be iterative and flexible thus allowing the voices of those not heard during this initial process to be captured subsequently. It is likely that some of those who attended also filled in the audit tool, the workshop provided an opportunity to elaborate on these responses. The responses were analysed thematically by the SG, and the identified themes were captured on the record card. See Table 1.

**Table 1:** A summary of 17 staff member and Advisory Panel responses to the audit tool

Recording key points from your discussion	
Values	Why do public involvement (PI)? To increase the relevance and improve the quality of research To ensure, the language used is appropriate and understandable across a range of audiences To influence policy To ensure, knowledge is shared/disseminated appropriately To give a voice to the “public”
Approaches to PI	Different ways that PI is undertaken currently Involvement in: Staff training Study design Intervention development and design Research as participants Recruitment of research participants Dissemination of results (in a variety of ways) As grant holders/co‐applicants
Practical issues	What wider influences have shaped PI work at Spectrum Centre? National bodies/research frameworks eg, INVOLVE Internet and social media Involvement of people with lived experience of bipolar disorder (service users, relatives and carers) in the Spectrum Centre What practical issues have shaped PI work at the Spectrum Centre? Problems with financial support for service users involved in different ways Trying to establish appropriate structures to support, manage and organize PI: Advisory Panel and Spectrum Connect Important that people are paid for their involvement but should not undervalue the importance of voluntary involvement as well. Rules governing welfare payments may impose some limits on the amount of work people are able to do
Identifying the impacts of PI in research	How does PI affect the research process and conclusions? Increases relevance Increases validity Increases credibility Reduces stigma What difference does PI make? Foregrounds the lived experience of bipolar disorder Opens a wider audience for the research How do you think PI could be assessed at the Spectrum Centre Questionnaires and feedback Monitor PI

##### Workshop

Following the administration of the audit tool, all members of the centre's staff and service users on the Advisory Panel were invited to a workshop to allow participants to elaborate upon their responses to the audit tool. After a brief introduction to PiiAF and its development, we facilitated a discussion based around the PiiAF record card, and new themes or ideas were incorporated into it. For the most part, workshop participants knew each other and were also experienced in PI in research. See Box 2 for a brief overview of the kind of issues that were raised.

BOX 2 **Issues that were raised in the follow‐up workshop that were additional to existing audit tool responses included**

**Values**

Public involvement (PI) was seen as a way of giving a voice to service users; as a means of making research more relevant and acceptable and as a way of reducing stigma associated with bipolar disorder.

**Approaches to PI**

The importance of PI happening throughout the research process and using a range of different approaches was highlighted.

**Research focus and study design**

Some aspects of the mental health research context were experienced as paternalistic, so the importance of PI in counteracting this was emphasized.

**Practical issues**

A range of practical issues including financial resources, the national research policy context and pathways of engagement with the Spectrum Centre were identified.

**Identifying impacts**

There were mixed messages about whether PI impacts had been assessed. One key impact that emerged was the importance of foregrounding lived experience of bipolar in research.

#### 3.1.2 Using Part 2 of PiiAF

Part 2 supports the development of an impact assessment plan, drawing on the information obtained during Part 1. The aim was to clarify the reasons for carrying out an impact assessment, understand how the PI approach will lead to the kinds of impacts that are intended and also to consider potential impacts that were not intended. To carry out Part 2 of PiiAF, we used a number of tables (see below), provided in the PiiAF guidance document, to carry out the recommended steps in the process.

##### Phase 1: Laying the foundations

We had discussed the reasons for assessing PI impact in the Spectrum Centre at early meetings of the SG. The reasons included to test PiiAF; to make the approach to PI in the centre more explicit; to generate ideas about how to develop the PI strategy within the centre; and to create an evidence base to support PI costs in funding applications. The SG agreed that all members of the centre should have an opportunity to contribute in some way to the process of designing the impact assessment plan; however, it is likely that, depending upon what the impact assessment focuses upon, smaller groups of Spectrum Centre members would be involved in conducting the actual impact assessment.

##### Phase 2: Developing your intervention theory

Using information obtained from other members of the Spectrum Centre through the audit tool and workshop SG members began the process of developing our intervention theory. First, we identified a number of potential impacts of PI on the work of the centre that could usefully be assessed:

1. PI impact on service user pathways for engagement with the Spectrum Centre.
2. PI impact on research agenda setting.
3. PI impact on the interpretation of findings.
4. PI impact on the dissemination of findings.

Once the SG had identified the PI impacts that we were potentially interested in exploring and, prompted by the framework, we thought about how the Spectrum Centre approach to PI could lead to the desired impacts. This led us to specify a number of intervention theories (see Table 2 below).

**Table 2:** Intervention theory and impact assessment plan

Intervention theory	Impact assessment plan
Public involvement (PI) impact on service user pathways for engagement with Spectrum Centre: PI through Advisory Panel members ensures more relevant service user involvement with Spectrum Centre by broadening the range of recruitment activities (through new ideas, new networks)	Record routes to involvement before and after the implementation of a recruitment strategy developed by Spectrum Centre Advisory Panel members
PI impact on research agenda setting: PI through service user researchers (SURs) ensures that research priorities are more relevant as a result of research project proposals being informed by lived experience of people with bipolar disorder	Explore similarities and differences in research priorities between Spectrum Centre academics and SURs. Use qualitative methods to explore perceptions of relevance
PI impact on the experience of participating in research: Employing SURs to collect data and making their status known to participants will make the experience of participating in research more positive for service users because of their shared experience and status	Record differences in service user participant experiences in a study where half experience interviewers who disclose their service user status and half do not. Use qualitative methods to explore the participants’ experiences
PI impact on the dissemination of findings: PI through Advisory Panel members ensures that findings are disseminated to wider and different audiences because of access to and understanding of wider and diverse networks	For a proposed/hypothetical study, compare the dissemination plans of: a group of academics only Advisory Panel members only a group comprising both academics and Advisory Panel members

##### Phase 3: Identifying the possible effects of context on the impacts of PI

Once the intervention theory or pathway between our approach to PI, specific methods for involvement and the desired impacts were identified the two members of the SG who work for the Spectrum Centre were asked to consider how the context in which PI in research takes place might influence its process and impacts and to capture this in writing. The SG was asked to consider how the context in which PI in research takes place might influence its process and impacts and to capture this in writing.

This exploration of context and its influence on PI process and impact suggested that some key features of PI in the Spectrum Centre would be likely to lead to positive impacts. PI

1. is embedded within the ethos of the centre;
2. is integral to or considered throughout the research process;
3. happens in different ways through SURs, the Advisory Panel, the establishment of project specific Service User Reference Groups, online and through a variety of outreach work and events.

Some potential barriers to maintaining PI were also identified especially around ongoing funding of PI. In addition, there was some concern about the wider institutional context, which currently does not fund PI as a matter of routine and organizational procedures that can make remunerating PI difficult. Other structural challenges include a lack of formally recognized or supported career structures for SUR or personal development strategies. It could be argued, however, that awareness of these potential barriers means that the centre can be an advocate for change in institutional processes and attempt to mitigate in a limited way the potential challenges faced by members of the public engaging in PI.

##### Phase 4: Formulating the assessment questions and designing the assessment

Phase 4 focuses upon identifying the specific impact assessment questions to be addressed and identifying the data that could be collected. The PiiAF guidance provides some tables that can help with this that are illustrated below. Table 3 contains the impact assessment questions we generated using a resource provided with the PiiAF guidance.^1^

**Table 3:** PI impact assessment questions

WHO?	HOW?	WHAT?
Public involvement (PI) impact on service user pathways for engagement with Spectrum Centre: Does PI	Through Advisory Panel members leading the development of Spectrum Centre recruitment pathways	Lead to an increase in the number and diversity of service users recruited to take part in Spectrum Centre activities?
*PI impact on research agenda setting*: Does involving service users	As SURs collaborating with academics on research priority development in Spectrum Centre	Lead to proposals that are perceived by key stakeholder groups to be more relevant and informed by lived experience of bipolar disorder?
*PI impact on the experience of participating in research*: Does involving service users	As SURs to collect data	Change the experience of participants taking part in studies?

Next, the PiiAF process invites research teams to consider how the intervention theory and research questions can inform decisions around impact assessment design and data collection. Table 4 below shows how we used the intervention theory and impact assessment plan and the impact assessment questions from Tables 2 and 3 to identify indicators, measures and sources of data.

**Table 4:** Elements of impact assessment plan for public involvement (PI) impact

State your intervention theory	Impact assessment plan	Impact assessment question	Identify indicators	Develop measures	Data
*PI impact on service user pathways for engagement with The Spectrum Centre*: PI through Advisory Panel members ensures more relevant service user involvement with The Spectrum Centre by increasing the range of recruitment activities (through new ideas, new networks)	Record routes to involvement before and after the implementation of a recruitment programme developed by The Spectrum Centre Advisory Panel members	Does PI through Advisory Panel members leading the development of The Spectrum Centre recruitment pathways lead to an increase in the number and diversity of service users recruited to take part in The Spectrum Centre activities?	Increased levels and diversity of service user involvement in The Spectrum Centre activities	Quantitative comparison of engagement pathways before and after recruitment programme eg, Iliffe, McGrath & Mitchell (2011)^18^	Quantitative‐retrospective records of service user involvement in The Spectrum Centre and prospective records of service user involvement
*PI impact on research agenda setting*: PI (through either/or SURs and Advisory panel members) ensures that research priorities are more relevant as a result of research project proposals being informed by lived experience of people with bipolar disorder	Explore similarities and differences in research priorities between Spectrum Centre academics and SUs. Use qualitative methods to explore perceptions of relevance by different groups	Does involving service users on research priority development in The Spectrum Centre lead to proposals that are perceived to be more relevant to key stakeholder groups?	Differences in research priorities proposed by SURs and academics	Ratings of relevance made by key stakeholder groups outside the research team including service users and grant funders	Qualitative—thematic coding of identified priorities (eg, Brown et al, 2006) Ratings of relevance from key stakeholder groups
*PI impact on the experience of participating in research*: Employing SURs to collect data and making their status known to participants will change the experience of participating in research	Record differences in service user participant experiences in a study where half are exposed to interviewers who disclose their service user status and half do not. Use qualitative methods to explore the participants’ experiences	How does involving service users as SURs to collect data change the experience for participants?	Service user satisfaction ratings with participation in a study comparing those exposed to disclosing SURs and those not Retention rates across these 2 groups	eg, Client Satisfaction Questionnire‐8 (Guarino et al, 2006) See also: Hamilton et al (2011)	Quantitative measures—responses to Client Satisfaction Survey and levels of recruitment and retention of participants exposed to the 2 conditions Qualitative interviews with participants to understand their experiences of taking part
*PI impact on the dissemination of findings*: PI ensures that findings are disseminated to wider and different audiences because of access to and understanding of wider and diverse networks	For a proposed/hypothetical study, compare the dissemination plans of: a group of academics only Advisory Panel members only Advisory Panel members only a group comprising both academics and Advisory Panel members	Does PI through Advisory Panel members’ collaboration with academics in developing dissemination plans lead to wider and more diverse dissemination activities?	Assessment of the number and range of outlets in which dissemination takes place Assessment of the number and range of activities undertaken	Quantitative & qualitative comparison of dissemination activities before and after Advisory Panel involvement eg, Van Staa et al (2010)	Quantitative data assessing number and range of activities, outlets, qualitative interviews with academics and Advisory panel members to check their understanding of the difference that PI makes to dissemination activities

As part of the development of PiiAF, a database of studies with detailed descriptions of their methods and the tools that were used to assess the impact of PI was produced.^3^ Using this searchable database, we were able to identify a number of existing studies that had explored some of the impacts we were interested in assessing.^18^, ^19^, ^20^, ^21^, ^22^ Being signposted to these studies helped us to think about how we could develop an impact assessment that engaged with the existing evidence rather than starting from scratch.

### 3.2 Critical Reflection on Key Moments of our use of PiiAF

In the section above, we have provided a detailed description of how we used PiiAF, we will devote the remainder of the paper to a critical reflection on this experience to identify learning that might be helpful to others who are considering using PiiAF. The following sections are structured according to the key moments in the PiiAF assessment process identified in the critical reflection template completed by the 4 members of the SG.

#### 3.2.1 Initial meeting

Reactions during this initial stage amongst SG members, especially those with no prior experience with PiiAF, included difficulty in understanding exactly what PiiAF was for and how PiiAF might work. For example, there was confusion initially about what the purpose of PiiAF was, with some SG members thinking it was to develop a plan for how to do PI rather than assessing the impact of PI. However, this lack of clarity was resolved as the SG became more familiar with the PiiAF documentation. We were also concerned that because the SG contained two members who were involved in developing PiiAF (MC, FL) our experiences might not reflect those of other research teams who were starting from a position of having no prior knowledge or experience of the framework. Although familiarity with the framework was definitely helpful in the initial stages, the process of applying the framework to a real‐world context was something new to all SG members.

#### 3.2.2 Audit Tool

Use of the audit tool developed by the SG and our subsequent identification of themes from the responses received from centre staff and members of the public involved in the centre's research were generally considered to have been useful. SG members could see that something tangible was happening and we could begin to understand how PiiAF would work. There was some discussion during the development of the audit tool about whether we should provide example answers as a guide. Our concern that they may bias the kind of responses we received led them to be omitted, but the potential benefits of helping respondents understand the task were noted.

#### 3.2.3 The workshop

Although the workshop was generally thought to have gone well and achieved its aim of gaining a greater understanding of the rather telegraphic data provided by the audit tool, there were challenges in running this. Public involvement is an emotive subject, and some of the participants (mental health service users) had powerful stories to tell, including at times, their own traumatic life experiences that led them to be in the position of being a service user. At times, the discussion was shaped by the need for people to share their experiences in detail, rather than addressing the questions posed. In the experience of RL, the SUR, this challenge was not unique to this context. The presence of RL, an established Advisory Panel Chair and SUR who knew both Advisory Panel members and staff well, helped to address this challenge by overtly acknowledging the needs of the participants, whilst also focusing their attention to the questions being addressed. Because of the sensitive nature of the topic, we felt that it was important to give people time and space to discuss or air any strong feelings evoked by the topic. It would have been inappropriate to close down such discussions to focus exclusively on the questions to be answered. We do not see it as problematic that such discussions took place, but rather consider it to have been part of the process.

#### 3.2.4 Updating the record card

The record card which we used to record the themes identified from the centre staff and members of the public involved in the audit process and the workshop (described above) worked well as a physical prompt and meant that we could capture responses to the Part 1 elements of PiiAF systematically. However, it was not always easy to keep in mind its role in developing the impact assessment plan. The record card did not inform our early discussions in Part 2 of the PiiAF process, and this potentially made those early stages more difficult. PiiAF is a flexible process, and for a whilst, the SG parked the record card responses to focus our attention on generating impact assessment questions, developing intervention theories etc. Returning to the record card at a later stage reminded us how our proposed impact assessments mapped onto the values and the impacts that had been made explicit in Part 1. This provided us with greater focus, but could easily have been missed as the link between the 2 parts of PiiAF was not immediately obvious to us.

#### 3.2.5 Working through Part 2

All phases of Part 2 were felt to make sense in their own right, but there was some concern about how Part 1 fitted with Part 2 and as a consequence we did not necessarily follow the order of the framework as set out in the guidance. However, the PiiAF guidance says that it can be used in any order and iteratively, so this was not a problem. For example, we discussed the purpose of the impact assessment and who should be involved (key elements of Part 2) during early meetings of the SG before we had even begun to engage with Part 1 of the framework. During Part 2, in particular, we felt that some of the terminology and concepts did not feel user‐friendly, for example, some SG members were unsure about what an intervention theory was. We identified a larger number of potential impacts to assess than we anticipated, and to a certain extent, this overwhelmed us. We also became distracted by thinking about how we might actually put the impact assessment plans into practice and tried to identify potential sources of funding. However, as we began to complete the tables to identify the assessment questions and data collection methods, the process began to feel more tangible and got back on track.

#### 3.2.6 Overall reflection on process

We found the process of using PiiAF useful. Part 1 was particularly helpful in surfacing the underlying and diverse values around PI held amongst members of the centre, encouraging us to identify how the context in which we were working enabled or constrained PI and making us aware of the wide range of potential PI impacts that could be assessed. Part 2 felt less tangible and user‐friendly initially and required a deeper engagement although we felt more confident with the process when we began to populate the tables to identify the intervention theory and develop the impact assessment plan. However, as the work involved in Part 2 requires some research expertise, it is perhaps not surprising that it is more challenging than the general discussions triggered by Part 1.

### 3.3 Discussion of our experiences of using PiiAF in the context of previous research

PI in research is characterized as a complex social activity.^1^, ^2^, ^3^ Attempting to develop an impact assessment plan for PI in a research centre rather than an individual research project adds an additional layer of complex and time‐intensive social activity—groups with a pre‐existing commitment to, and valuing of, genuine and effective PI in research will perhaps be more willing to make this time available. This might account for the well‐documented difficulties associated with developing a sophisticated evidence base.^2^, ^3^, ^9^, ^23^

As PI in research is so firmly located within multiple interpersonal, social, research and cultural contexts (eg, that of members of public involved, the researchers, the wider community, funders) it is unsurprising that there have been calls to make greater links between process and context variables and PI impacts.^2^, ^3^ The use of the PiiAF record card in Part 1 to capture key points from the discussions of the contextual complexity and the development of an intervention theory in Part 2 (although difficult conceptually) that made explicit our thinking about how the PI process would lead to the hoped‐for impacts supported us in making exactly these kinds of links between process and context and PI impact. In our experience, PiiAF represents a way of negotiating the complexity without making the process of assessing impact so simple that it lacks validity or sensitivity to the specific context in which it is being used.

By not being prescriptive about the kind of evaluation design that research teams should use when developing an impact assessment plan, PiiAF supports the development of an elaborated understanding of the variables that will make positive impacts of PI in research more likely.

The backgrounds of the members of the SG variously included service user research, clinical research and social constructionist qualitative research, and we feel that by using PiiAF, we would have been able to develop a plan that would have been acceptable within any of these paradigms.

### 3.4 Future action—Some Insights from using PiiAF

Research teams will find their own ways of using PiiAF that will be contingent upon the particular context of their PI and their research, their expertise, resources and time availability. However, there are some things that we have learnt from using PiiAF that other research teams might find useful and we set out 10 insights below. It is important to note that these are based on one case example of our experience of using PiiAF. They are offered in the hope they will stimulate further exploration of the process of developing ways to assess the impact of PI. They do not necessarily reflect the views of the team that developed PiiAF.

1. PiiAF is a complex process rather than a quick‐fix measure, but this allows greater flexibility and is more likely to lead to effective impact assessment plans.
2. The context in which we used PiiAF was important. The Spectrum Centre already had PI systems and processes in place that we could draw on to implement the stages of using PiiAF. Research teams that do not have this will need to consider how they will support PI and involve members of the public in the planning and/or implementation of an impact assessment. It is possible to use PiiAF without a supporting infrastructure for PI in place, but the process is likely to take longer whilst time is taken for recruitment of members of the public and protocols for working are established.
3. We had decided at an early stage that PI in the development of the impact assessment plan was important both ideologically (in keeping with the philosophy of The Spectrum Centre and PiiAF) and practically (ensuring access to information for and the credibility of the impact assessment). [Information redacted to preserve anonymity]. The SG consisted of two members of Spectrum and two people independent of it and this was a helpful combination. The 2 Spectrum Centre members had good understanding of PI in the Spectrum Centre; they facilitated access to meetings and had knowledge of context, which we were able to draw on when filling in the record card. One of the SG members was an experienced SUR involved in the process who was able to bring her experience, expertise and act as a link between members of the Spectrum Centre team and service users who were involved on a sessional basis. The 2 SG members independent of The Spectrum Centre received and analysed audit tool responses and supported the running of the workshop, which created a certain amount of distance and perceptions of impartiality. We recommend that research teams think carefully about who is involved in organizing the impact assessment.
4. We did not always do the tasks of PiiAF in the order presented, but the guidance does clearly state that users can use it in any order and iteratively. We did tasks as they cropped up in our discussions or when it made sense to look at them. For example, we had discussions about the purpose of the impact assessment and who would be involved in the first meeting of the SG, but the PiiAF guidance suggests that this discussion would be best taking place at the beginning of Phase 2. This did not seem to cause any problems in the process, and we suggest that research teams use PiiAF flexibly as indeed it was designed to be used.
5. Filling in the record card with key points arising from discussions prompted by the PiiAF questions was a helpful process in and of itself. Research teams that are interested in improving the quality of their PI without necessarily assessing its impact might find it useful to go through Part 1 along with the PiiAF Draft Standards for Good Practice^4^ in PI resource on the PiiAF website.
6. We found it quite a difficult process to use the record card in Part 2 and would in retrospect like to have taken more time to think about how our learning from Part 1 could be linked to the challenges we had to address in Part 2. Part of the challenge was that we generated a lot of ideas about the kinds of impacts we would like to explore. This may not be a consequence of using PiiAF and may have happened in any focused discussion of likely impacts. Given the subsequent PiiAF stages of developing intervention theories and impact assessment plans, we went through a process of narrowing our focus on a small number of impacts, but it would be helpful to have some practical guidance about how to prioritize ideas. As we were not in a position to implement an impact assessment plan, reducing the number of impacts to be assessed was a hypothetical question for us. We included most of the impacts that we identified in the figures presented earlier to demonstrate the different kinds of impacts that could be assessed to maximize the utility of this paper as a learning resource. The small amount of narrowing down we did was informed by the values and impacts that we had made explicit in Part 1 of the process of using PiiAF. We suggest that research teams factor in time to think about how they might identify which impact assessment ideas they would like to take forward.
7. We struggled with intervention theory both conceptually and practically and seemed to spend a lot of time on this. PiiAF understands this difficulty and that it requires research expertise and provides some resources to support this. After going through a process of reflection, we recognize that the intervention theory is potentially crucial to making explicit the context and involvement process and their links with impacts. Although perhaps not immediately intuitive, it is worth persevering with this step in the process to ensure that these links are made and also to check that the PI approach is appropriate to the desired impacts.
8. During development of the impact assessment plan, we encountered problems conceiving plans that would allow us to attribute impact to PI (rather than anything else) especially given the type of research carried out in The Spectrum Centre which tends to be multidisciplinary, pragmatic research employing a wide range of methodologies. This, as already discussed, is a challenge common to PI assessment and was made even more challenging because The Spectrum Centre already has an established programme of PI activity, making it difficult to identify ways to compare research processes that did involve PI with those that did not. Whilst PiiAF did not provide any easy answers to this, it helped us to identify some of the challenges.
9. PiiAF was developed by a multidisciplinary team with a strong social science background and is primarily aimed at research teams that include professional researchers. All of the members of our team struggled to understand some of the terminology, for example, as discussed “intervention theory,” “normative values.” Therefore, from the outset, it is helpful to establish a shared understanding of some of the key words and concepts in PiiAF and encourage people to ask for clarification regarding any terms that are not familiar to them. The PiiAF guidance^1^ contains a helpful glossary of terms.
10. Groups that have less familiarity or experience of PiiAF might find it useful to use some of the resources developed for PiiAF that can be found on the PiiAF website.^5^ For example, to build on the existing evidence base rather than start from scratch each time, it is important to engage with the existing literature on impacts. The Impacts Database helped us to identify studies that may be relevant to our impact assessment during phase 4.

## 4 CONCLUSION

The aim of this paper was to provide a case example of using PiiAF to develop an impact assessment plan for PI in a mental health research centre. We chose to use a reflective case study so that we could share key aspects of our experience and the practical steps involved. We have shared what we learnt from this process in the hope this may be of use to other teams considering the use of PiiAF and also those who are unfamiliar with it or had not thought about using it. The insights gleaned from this process presented above need to be considered alongside the limitations of our study design.

Firstly, we were using PiiAF in a different way than was originally intended—in the context of a mental health research centre considering its existing PI, rather than during the planning stage of a single research project or programme. We feel that the flexibility built into PiiAF meant that we were able to apply it, albeit with some minor adjustments in this context. Secondly, our SG contained people who were already familiar with PiiAF so we perhaps did not use the Piiaf guidance as fully as we might. However, we think the guidance will be invaluable to those who are less familiar with the PiiAF as it includes key questions to consider, case examples and a glossary of key terms. Thirdly, alongside contemporaneous meeting notes our case study relied on retrospective recollections that are notoriously open to well‐documented biases and distortions. We might not have completely captured what people felt at each stage of the process. In future, it would be helpful to facilitate contemporaneous reflections by building in documented reflection to the study protocol.

That we were able to produce a detailed PI impact assessment plan suggests that PiiAF was useful for us; it gave us a focus for meetings and provided us with a place to start. The website associated with PiiAF contains a range of resources that were helpful. The process was not a straightforward one and required a commitment of time and effort to engage with the concepts and materials. We feel that for us, this was worthwhile in helping us articulate motivations for PI at The Spectrum Centre, clarifying the PI strategy and developing several PI impact assessment plans. Our next step is to implement these plans and evaluate the impact of PI in our work.

## CONFLICT OF INTEREST

Three of the authors (MC, JP and FL) were part of the original research team who developed the PiiAF.

## Notes

1
http://piiaf.org.uk/

2 To register with Spectrum Connect connect go to http://www.lancaster.ac.uk/shm/research/spectrum/spectrumconnect/

3
http://www.piiaf.org.uk/documents/impacts-database.pdf.

4 Accessed at: http://piiaf.org.uk/documents/piiaf_draft_standards.pdf

5 Accessed at: http://piiaf.org.uk/resources.php

## APPENDIX 1

### Initial protocol discussed at first meeting of the Steering Group for using PiiAF within The Spectrum Centre

**Table:**

How will the activities associated with these 2 parts be achieved Part 2: What will the impact assessment plan look like and who will develop it?	We anticipate that: Part 1: To collect information about public involvement (PI) values, anticipated impacts, PI approach we will use regular steering group discussions, including mostly face‐to‐face but possibly telephone. We will liaise with members of the centre to obtain information. Frequency of SG meetings to be 3 or 4 weekly initially but will adjust according to the demands of the project Part 2: To develop the impact assessment plan. Work will be focused within the SG—assess the need for frequency of meetings
How will the impact assessment plan be implemented?	To implement the assessment plan, we will need to identify sources of funding. The feasibility of this will be assessed as the project proceeds
Will we evaluate the PiiAF‐based impact assessment?	This will depend on obtaining funding (above)
Who will be involved (small group, whole team?) Developing the impact assessment plan Carrying out the impact assessment Evaluating the impact assessment?	Initially, the SG will be involved in pulling together the information needed to develop an impact assessment plan. Part 1 will involve centre members of staff and service users in providing their responses to. Developing the impact assessment plan (Part 2 of PiiAF process) will involve SG members only. Carrying out/evaluation impact assessment will depend upon obtaining funding
How long will it take?	This is hard to gauge. There is no funding for this project, so depends upon the availability of SG members to both attend meetings and undertake pieces of work outside of the meetings. Aim for 6 mo initially but review as the project progresses

## APPENDIX 2

### Record card completed iteratively from audit tool responses, workshop discussion and experiences of Spectrum Centre members on the SG

**Table:**

Recording key points from your discussion	
Values	Why do public involvement (PI)?: To increase the relevance of research *to service users and funding bodies* To improve the quality of research To ensure, the language used is appropriate and understandable To influence policy To ensure, knowledge is shared/disseminated To give a voice to the “public” (especially people with lived experience) *but not about being representative (people with bipolar not an homogeneous group) and about doing research with but not on people* To reduce stigma—*to challenge stigma through both the content (research topics) and the process (people with bipolar having an active involvement in the research process). Ways in which stigma is challenged include getting paid/being successful in society/diversity of role models/being an ambassador/being listened to* How does PI affect the research process and conclusions?: Increases relevance Increases validity Increases credibility Reduces stigma
Approaches to PI	Important that involvement happens throughout the research process: Staff training—*interview training for researchers—makes the interviews more accurate/sensitive, realize how it is for the person being interviewed* Study design Intervention design As research participants In the recruitment of research participants *and in the recruitment of researchers* In the dissemination of results (in a variety of ways) As grant holders/applicants
Research Focus and Study Design	Some involvement is informed by peoples’ experiences of mental health professionals and the mental health system. Perception that mental health system can be infantilizing or paternalistic—important that involvement counteracts this( )Mental health system/professionals also influence recruitment to studies and can act as gatekeepers. In turn, this shapes the impact that PI can have in that a SUR representing the study makes it more credible than if researchers represent it
Practical Issues	What wider influences have shaped PI work at The Spectrum Centre?: National bodies/research frameworks Internet and social media Involvement of people with lived experience of bipolar disorder (service users, relatives, carers) in The Spectrum CentreWhat practical issues have shaped PI work at The Spectrum Centre?: Problems with financial support for service users involved in different ways Advisory panel ‘Spectrum Connect’—*the nature of funded grant research can cause difficulties because there is no money to keep people going between projects and grants* Important that people are paid for their involvement but should not undervalue the importance of voluntary involvement as well. Rules governing benefits may impose some limits on the amount of work people are able to do *Routes into involvement—The Spectrum Centre offers a range of opportunities but people need to approach the centre first* *Social media as a way of raising profile of involvement in The Spectrum Centre—but also be aware of those excluded by this method of awareness raising* *Importance of providing feedback to ensure that cpns continue to engage with the study and support recruitment*
Identifying the Impacts of PI in Research	What difference does PI make?: Foregrounds the lived experience of bipolar disorder*—acts as a reminder that research is studying people and not a disease. For example, the focus on anxiety in the Parades study* Opens a wider audience for the research—*wider dissemination opportunities* *Keeps you on your toes* Assessment of PI: *Was the impact of PI assessed? How?* No or not sure Social media *Qualitative interviews with people who have participated in studies shows the value of PI impact* *How do you think PI could be assessed at The Spectrum Centre*?: Questionnaires and feedback Monitor PI *This meeting and the system/ethos that allowed it to take place is an example of PI impact*

## APPENDIX 3

### Critical reflection template used by SG members

Critical reflection template.

The table below contains a timeline of activities and meetings since the project began in January. As part of our mapping of the process of using PiiAF, it would be helpful if you could fill in any reflections on these activities for example.

- what worked well
- what did not work well
- how well you understood this part of the process
- how well you understood how this activity was linked to the design of an impact assessment plan
- whether you can think of a better alternative

**Table:**

Date/activity	Reflection
Date: initial meeting to discuss the project	
Date: audit tool sent out to staff and Advisory panel	
Date: meeting to discuss the themes identified from the survey responses—we filled in the record card using these themes	
Date: workshop to elaborate on the audit tool responses	
Date: meeting to de‐brief after the workshop, we updated the record card to include workshop responses	
Date: meeting to discuss Part 2 activities	
Date: description of activity	
